# Dermatophyte infection presenting as flat topped/lichenoid papules: An unusual presentation

**DOI:** 10.1016/j.jdcr.2025.05.032

**Published:** 2025-07-14

**Authors:** Lamia AlAkrash, Alanoud A. AlSalman, Iman I. Nazer, Salman Al Malki

**Affiliations:** a Department of Dermatology, King Fahad Medical City, Riyadh, Saudi Arabia; b Department of Dermatology, Security Forces Hospital, Riyadh, Saudi Arabia; c Department of Pathology, King Fahad Medical City, Riyadh, Saudi Arabia

**Keywords:** dermatophyte infection, dermatophytosis, itraconazole, lichenoid papules, tinea corporis

## Introduction

Dermatophytosis are superficial fungal infections of the skin, hair, or nails caused by the filamentous fungi known as ringworm or tineas.^1^ The clinical features of dermatophyte infection depend on the causative organism and the host immunity; they can last for months or years, and infected individuals can be asymptomatic or have solely pruritus.^2^ Tinea corporis, also called ringworm of the body, has a characteristic morphology, which is well defined round annular pink patches with raised edges and central clearing.^3^ Atypical presentations of tinea corporis reported in the literature include: targetoid bullous lesions over the neck, trunk and forearm, subcutaneous nodules/abscesses over the lower extremities and erythematous papules/plaques over the back and posterior arms of a neonate.^4^, ^5^, ^6^

We present a 14-month old boy with unusual numerous dermatophytic lichenoid-like papules over the back.

## Case report

**Fig 1:**
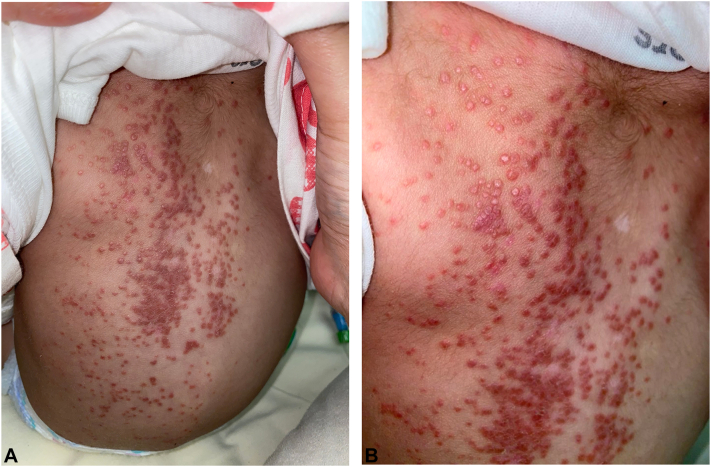
**A** and **B,** There are numerous *pink* and *red-brown* flat-topped papules over the back, coalescing into faintly scaly plaques.

A 14 month old full term boy, known to have congenital hydrocephalus on ventriculoperitoneal shunt was admitted to the hospital for ventriculoperitoneal shunt infection, for which he received 15 days of cefepime and vancomycin. Four days after admission he developed numerous papules over the back. There was no history of contact with sick patients or animals. Examination revealed numerous pink and red-brown flat-topped papules over the back, some coalescing into faintly scaly plaques (Fig 1). Darier’s sign was negative. The laboratory investigations revealed an unremarkable complete blood count/differential except for low white blood cell count 4.57 10∗3/uL [N:>6.00-<18.00 10∗3/uL], high relative neutrophil count 42% [N:>20-<35%], and hepatic and renal function tests were unremarkable.

**Fig 2:**
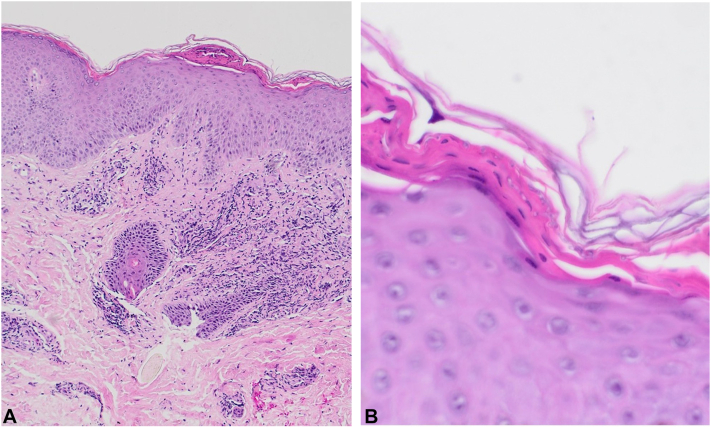
Microscopic examination of the skin punch biopsy showed epidermal spongiosis with focal parakeratosis, and fungal spores and hyphae in the stratum corneum under low magnification ×10 **(A)**, and high magnification ×65 **(B)**.

**Fig 3:**
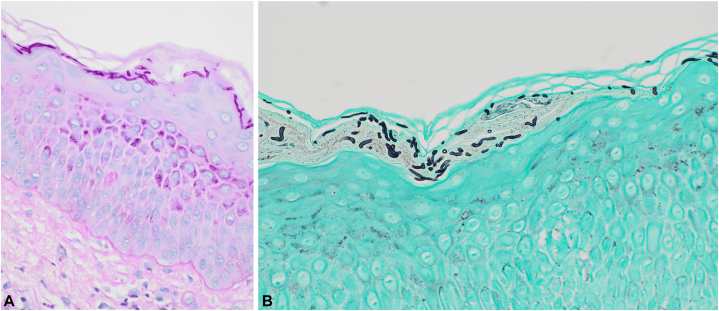
The photomicrograph shows fungal hyphae and spores in the stratum corneum after staining with periodic acid schiff (PAS) **(A)**, and grocott methenamine silver (GMS) **(B)**.

**Fig 4:**
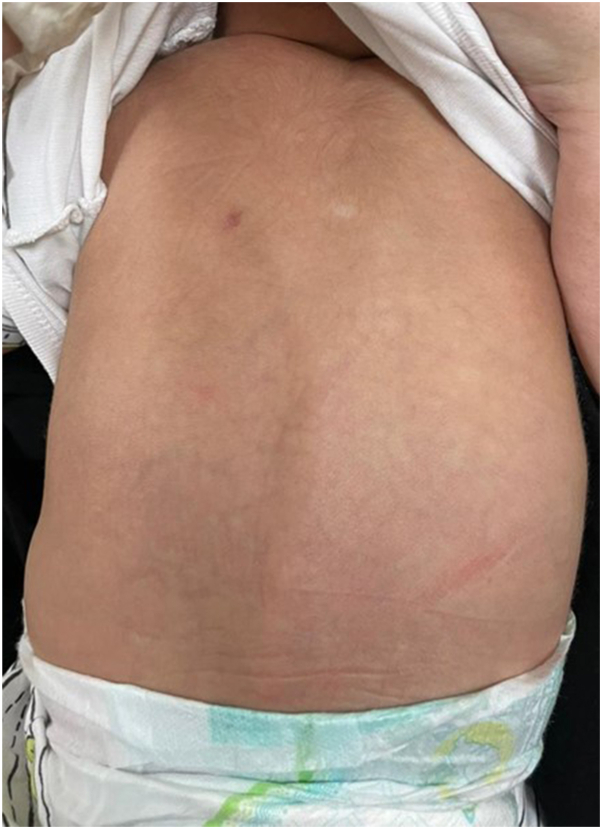
The photograph shows complete clearance over the back.

The clinical differential diagnoses considered were cutaneous candidiasis, flat warts, lichen planus, urticaria pigmentosa, and langerhans cell histiocytosis. A skin punch biopsy taken from the back showed epidermal spongiosis with focal parakeratosis, and fungal spores and hyphae in the stratum corneum, after staining with hematoxylin and eosin, periodic acid schiff and grocott methenamine silver stains (Figs 2 and 3) consistent with dermatophytosis. The patient was treated effectively with oral itraconazole, 5 mg/kg (50 mg) daily for 4 weeks (Fig 4).

## Discussion

We present a case of dermatophyte infection in a 14-month-old boy on broad spectrum antibiotics having the unusual presentation of numerous lichenoid papules over the back. Lichenified papules/plaques is an unusual presentation of dermatophyte infection, which was also reported by S Dekio in a 57-year-old male presenting with scrotal lichenified plaques.^7^

The development of nosocomial fungal infection following antibiotic use has been well-documented. As antibiotics directly affect both the metabolic processes in immune cells and the commensal organisms, this opens the body up for opportunistic infections. This is often observed to manifest as invasive candidiasis of the skin and gut, commonly attributed to Candida albicans. While the use of systemic antibiotics was justified in treating ventriculoperitoneal shunt infection, broad spectrum antibiotics are known to increase susceptibility to invasive fungal infections and increase morbidity and mortality. These effects may have had a role in this case as there was no history of high-risk contact.^8^

Given its similarities with other skin diseases, treatment for dermatophyte infection should only be started after an accurate and confident diagnosis. Additionally, misdiagnoses can lead to undesirable effects where diseases may progress, invade, and disseminate. Therefore, a decision was made to ascertain the diagnosis through skin biopsy.^9^

Suspected dermatophyte infections are either treated with topical or systemic antifungals. When infections are more widespread and severe, or not treated effectively by topical treatments, they may require escalation to systemic antifungal.^10^ Our patient was treated with itraconazole as he had an atypical presentation with widespread distribution all over the back.

In conclusion, we present a case of 14-month old boy with unusual presentation of dermatophyte infection presenting as flat topped/lichenoid-like papules over the back managed by systemic itraconazole.

## Conflicts of interest

None disclosed.
